# Development of Conductive Boron-Doped Diamond Electrode: A microscopic, Spectroscopic, and Voltammetric Study

**DOI:** 10.3390/ma6125726

**Published:** 2013-12-06

**Authors:** Kevin E. Bennet, Kendall H. Lee, James N. Kruchowski, Su-Youne Chang, Michael P. Marsh, Alexander A. Van Orsow, Aurelio Paez, Felicia S. Manciu

**Affiliations:** 1 Division of Engineering, Mayo Clinic, Rochester, MN 55905, USA; E-Mails: bennet.kevin@mayo.edu (K.E.B.); kruchowski.james@mayo.edu (J.N.K.); vanorsow.alex@mayo.edu (A.A.O.); 2 Department of Neurologic Surgery, Mayo Clinic, Rochester, MN 55905, USA; E-Mails: lee.kendall@mayo.edu (K.H.L.); chang.suyoune@mayo.edu (S.-Y.C.); marsh.michael@mayo.edu (M.P.M.); 3 Department of Physics, University of Texas at El Paso, El Paso, TX 79968, USA; E-Mail: apaez@miners.utep.edu

**Keywords:** confocal Raman microscopy, scanning electron microscopy, infrared absorption spectroscopy, fast-scan cyclic voltammetry, boron-doped diamond

## Abstract

Building on diamond characteristics such as hardness, chemical inertness and low electron emission threshold voltage, the current microscopic, spectroscopic and voltammetric investigations are directed towards improving the properties of electrode coating materials for their future use in clinical studies of deep brain stimulation via fast-scan cyclic voltammetry (FSCV). In this study we combine the capabilities of confocal Raman mapping in providing detailed and accurate analysis of local distributions of material constituents in a series of boron-doped polycrystalline diamond films grown by chemical vapor deposition, with information from the more conventional techniques of scanning electron microscopy (SEM) and infrared absorption spectroscopy. Although SEM images show a uniform distribution of film crystallites, they have the limitation of being unable to differentiate the distribution of boron in the diamond. Values of 10^18^–10^21^ atoms/cm^3^ of boron content have been estimated from the absorption coefficient of the 1290 cm^−1^ infrared absorption band and from the 500 cm^−1^ Raman vibration. The observed accumulation of boron atoms and carbon sp^2^ impurities at the grain boundaries suggests that very high doping levels do not necessarily contribute to improvement of the material’s conductivity, corroborating with voltammetric data. FSCV results also indicate an enhanced stability of analyte detection.

## 1. Introduction

Deep brain stimulation (DBS), as a state-of-the-art neurosurgical intervention, has been hypothesized to work by modulating neuronal activities, which may be caused by neurotransmitter changes [1,2,3]. Present DBS devices use an open-loop technique that delivers periodic electrical pulses without any internal feedback adjustment. Therefore, the development of a smart DBS system with feedback operation has generated great attention and interest. In order to develop a closed-loop DBS system, the long term and stable monitoring of neurotransmitter levels is critical. Diamond has unique electrochemical properties that make it ideal for this application since diamond electrodes are highly resistant to surface biofouling and chemical degradation, which has often been observed in carbon-fiber based microelectrodes. Long-term application of triangular voltage acquisition protocols for fast-scan cyclic voltammetry (FSCV) measurement degraded over 80% of the carbon fiber.

Conductive diamond electrodes and devices for chemical, high frequency and high power electronic applications with low and stable voltammetric and amperometric background current have been extensively studied from structural and optical perspectives [4,5,6,7,8,9,10,11,12,13,14,15]. Of all the potential dopants such as phosphorus, nitrogen, lithium, and sulfur, boron has been most commonly used because of its low charge carrier activation energy (e.g., the ionization energy of boron is about 370 meV [4] *versus* 600 meV for phosphorous and 1.6–1.7 eV for nitrogen [7]) and the compatibility of its covalent radius with that of the carbon atom (e.g., 0.088 nm for boron *versus* 0.077 nm for carbon). It has also been used as a co-dopant to promote incorporation of other dopants such as sulfur [7]. In FSCV bio- and electro-chemical sensing technology, besides boron-doped diamond’s (BDD) steady-state voltammetric response and low capacitance, its very high potential for both oxygen and hydrogen augments its viability as an electrode coating material [16]. Other coating materials made of chemically inert noble metals such as platinum and gold, at high oxidation or reduction potentials, failed to provide accurate detection of various analytes in solutions due to the high Faradaic currents produced in such reactions.

As boron, a trivalent atom, substitutionally incorporates into the diamond lattice, it leaves one uncompensated bond with the neighboring tetravalent carbon atom, causing p-type conductivity in BDD. Incorporation of this shallow electron acceptor at various concentrations has been reported [4,5,6,7,8,9,10,11,12,13,14,15,16]. While a low concentration is desirable for avoiding defects and imperfections in the doped material, a high concentration becomes more advantageous if metallic-like conduction is sought for electronic devices. However, it is worth pointing out that even at very high boron doping levels of more than 10^2^^0^ atoms/cm^−3^, conduction through hopping occurs instead of metallic conduction [17]. Thus, a careful balance of these two aspects should be thoroughly considered if improving material quality is envisioned, along with different conduction mechanisms that depend on boron concentrations. At low doping levels (*i.e.*, B atoms < 10^17^ cm^−3^) conduction through holes in the valence band is expected. With increasing boron concentration, a spread in the energy of the localized states of boron impurity centers induces the formation of an impurity band (with a band width of ~0.2 eV for a boron concentration of ~10^2^^0^ cm^−3^) and hopping conduction through nearest-neighbors or more distant impurities (variable range hopping) takes place [17,18]. At high doping levels, if not incorporated into the diamond lattice, boron and other non-diamond materials such as unwanted sp^2^ type impurities have been theoretically predicted to accumulate at the grain boundaries [19]. A drop in mobility was observed at these doping levels, especially for smaller grain crystallite sizes, where an enhancement of grain boundary effects is more likely [18,19]. Defects induced by substrate pre-treatment prior to film growth was another reported reason for this mobility drop [19,20]. The commonly used technique of abrasion with nanodiamond grits, while it increases the initial nucleation density and the successful growth of nano-structured films, could also create defects that limit the scattering mechanisms and, consequently, the film conductive properties. Thus, a complex combination of different effects including boron doping level, crystallite sizes (grain boundary effects), and existence of other unwanted defects and impurities contribute to material quality, affecting its performance in particular technological applications.

In investigations of carbon-based materials, the non-destructive Raman technique has been used as a main diagnostic tool, owing to the broad range of information it can provide in monitoring the perfection or the existence of point defects in diamond, in estimating inherent stress in grown material, and in semi-quantitatively measuring doping levels and annealing defects [5,21,22,23,24,25]. However, the majority of previous Raman studies of polycrystalline synthetically grown BDD films with grain sizes varying from nano- to micro-dimensions have been restricted almost exclusively to spectral analysis. Since confocal Raman mapping also facilitates direct visualization of the local distribution of film constituents (e.g., pure diamond, boron-doped diamond, and the existence of other unwanted impurities), the analysis presented in this work, which is complemented by information from infrared (IR) absorption and scanning electron microscopy (SEM), provides a comprehensive assessment of material morphological progression as a function of boron doping level. This information is essential if FSCV is envisioned, since understanding phenomena such as conductivity strongly depends on it. Although the factors that control and influence different BDD growth processes (the majority using highly toxic diborane as a precursor) and the electrochemical responses of synthesized materials for particular reaction mechanisms have been amply reviewed [5,11,12,13,14,15,18], the degree to which the physical, chemical, and electronic properties are interdependent still remains a challenge for scientists, as it varies from case to case.

## 2. Experimental Section

### 2.1. Fabrication of Boron-Doped Diamond Thin Films

In the current work, the BDD films were grown in a hot filament chemical vapor deposition (CVD) reactor using a mixture of CH_4_/H_2_ gases at a pressure of 20 Torr. Trimethylborane (TMB) at various partial pressures was introduced in the chamber to provide the necessary film conductivity. The TMB gas mixture was metered using an Alicat mass flow controller, and the H_2_ and CH_4_ gases were metered using FloCat flow controllers. The thin BDD films were grown on undoped single crystal (100) Si substrates or on tungsten thin rods that were initially abraded by immersion in an 8 nm diamond powder/isopropyl alcohol slurry mixture. The filament temperature was kept constant during film deposition and monitored with a Spectrodyne DFP 2000 optical pyrometer (Spectrodyne Inc., Perkasie, PA, USA). An Omega Engineering type K thermocouple was used to monitor the substrate temperature. All the parameters employed in the growth process of the thin films studied here are summarized in Table 1.

**Table 1:** Growth conditions used for boron-doped diamond thin films.

Sample number	CH_4_ (sccm)	H_2_ (sccm)	TMB (ppm)	P (Torr)	T (°C)	Power (W)	Growth time (h)	Thickness (μ)	Growth rate (μ/hr)
1	2	200	0	20	623	368	10.4	1.5	0.144
2	2	200	2.5	20	625	369	10.4	1.7	0.163
3	2	200	4.9	20	628	371	12.3	2.8	0.228
4	2	200	7.4	20	648	385	11.7	3.3	0.282
5	2.25	225	8.7	21	723	490	9.6	4.3	0.448
6	2	200	10.8	18	672	423	11.9	8.8	0.739
7	2	196	12.5	20	751	485	12.2	9.0	0.388

### 2.2. Microscopy and Spectroscopy Systems

SEM images were acquired with a Hitachi S4700 SEM (Hitachi Ltd., Tokyo, Japan), with accelerating voltage and emission set to 3 kV and 15 μA, respectively. Confocal Raman microscopy was performed with an *alpha 300R WITec* system (WITec GmbH, Ulm, Germany), using the 532 nm excitation of a Nd:YAG laser and a 100X objective lens with a NA = 0.90. The *WITec* Control software was employed for data acquisition and for controlling the piezoelectric stage for sample scanning. To obtain the Raman mapping images, the Raman signal was detected by a 1024 × 127 pixel peltier cooled CCD camera with a spectral resolution of 4 wavenumbers. A complete Raman spectrum was recorded in milliseconds at each pixel and the duration of the overall Raman mapping was on the order of minutes. Whereas Raman measurements were acquired at ambient conditions, a vacuum-based *Bruker IFS 66v* FT-IR spectrometer (Bruker Corp., Billerica, MA, USA) equipped with a KBr beamsplitter and a deuterated triglycine sulfate (DTGS) detector was employed for IR absorption spectroscopic experiments. Each IR spectrum resulted from an accumulation of 256 scans at a resolution of 4 cm^−1^.

### 2.3. Fast Scan Cyclic Voltammetry System 

FSCV measurements were performed with a Wireless Instantaneous Neurochemical Concentration Sensor (WINCS), which is an in-house sensing device developed at the Mayo Clinic in Rochester, Minnesota. The device’s detailed description can be found elsewhere [26]. A triangular voltage waveform ramping from −0.4 V to +1.5 V and back to −0.4 V was applied through an electrode every 100 ms at a rate of 400 V/s. The electrode was held at the baseline potential of −0.4 V between scans. An Ag/AgCl electrode was used as reference. Dopamine (DA) was injected through an *in vitro* flow injection system (FIAlab Instruments, Bellevue, WA, USA), and oxidation and reduction currents were detected at +0.8 V and −0.2 V, respectively. Tris-based buffer solution (in mM; 12 Tris-HCl and 150 NaCl) was used for the flow injection analysis.

## 3. Results and Discussion

### 3.1. Structure by Scanning Electron Microscopy

**Figure 1:**
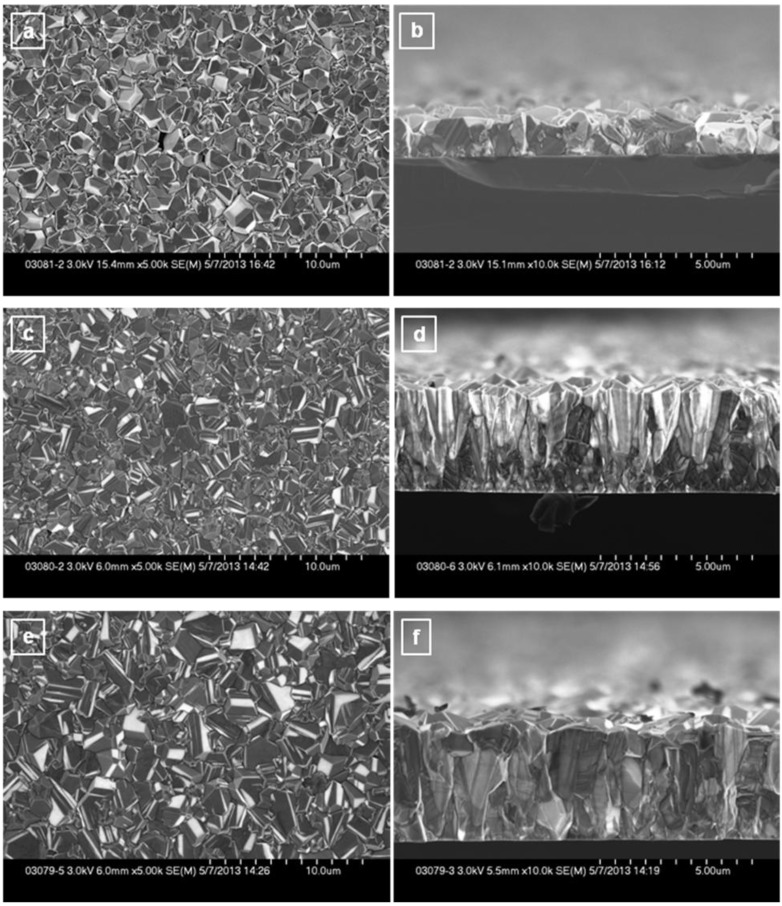
Top view and side-wall SEM images of: (**a**,**b**) undoped diamond (0 ppm TMB); (**c**,**d**) boron-doped diamond grown with 4.9 ppm TMB feed gas; and (**e**,**f**) boron-doped diamond grown with 7.4 ppm TMB feed gas.

Representative top view SEM images and their corresponding side-wall images for undoped and BDD films are shown in Figure 1a–f. The undoped film was grown under the same conditions, but without TMB addition. Since it is known that boron leaves residues on the walls of the reactor chamber and its other components, the chamber was thoroughly cleaned before performing the deposition of this sample, to avoid any unwanted boron doping content. The SEM image of this sample (Figure 1 a) reveals a relatively uniform film with crystallite sizes varying between 0.3 and 2.0 microns.

A very slight reduction in the average dimension of crystallites and an obvious increase in the growth rate induced by the addition of TMB feed gas can be seen in Figure 1c,d, respectively. An explanation of this previously observed and reported growth behavior is the fact that at relatively low concentrations of dopant, the overall chemistry is not highly disturbed and the methyl radicals (CH_3_) still play the important role in enhancing the nucleation and the growth rate of the species [23]. Thus, the grain size decreases as the growth rate increases with higher fluxes of methyl radicals (the TMB boron precursor used in this work contains methyl radicals, too). A slight increase in the average dimension of crystallites, ranging in size from 0.4 to 2.4 microns is observed with an additional increase in the amount of boron-containing gas to 7.4 ppm TMB (Figure 1e).

### 3.2. Confocal Raman Mapping Analysis

**Figure 2:**
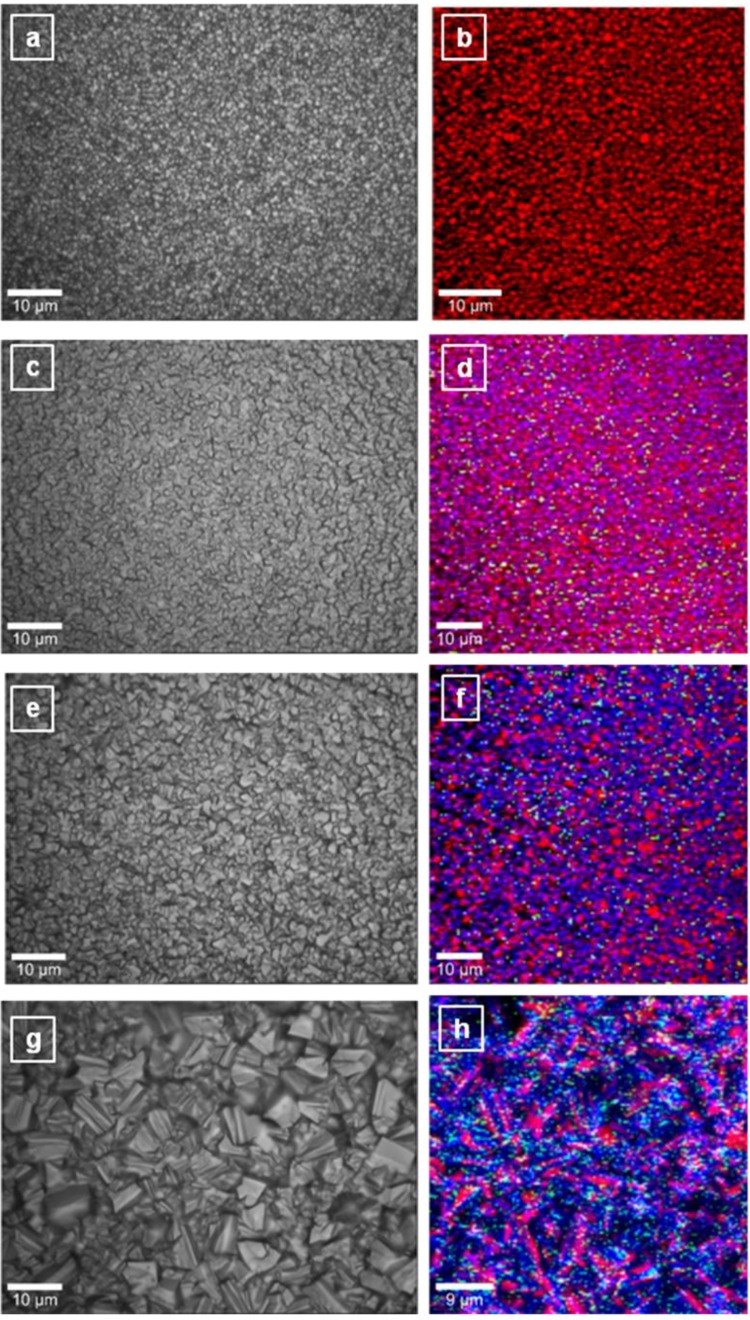
(**a**,**c**,**e**,**g**) optical images; and (**b**,**d**,**f**,**h**) corresponding surface Raman mapping images of undoped (0 ppm TMB) and boron-doped diamond samples prepared with 2.5, 7.4, and 10.8 ppm TMB. Red, blue, and green pseudo-colors are used for diamond, boron, and amorphous carbon impurities, respectively.

While SEM, which is the most frequently applied method of observing characteristic material microstructure, has the advantage of direct, high resolution imaging, it also has the limitation of being unable to directly reveal the precise spatial distribution of boron in the diamond structure. Since spectroscopy provides information about material composition at the molecular level, as seen in Figure 2a–h, confocal Raman mapping gives, simultaneously, imaging of the local distribution of pure diamond, boron doped diamond, and C sp^2^ impurities. Insights of this nature are particularly valuable for analysis of non-uniform materials. Optical images were also taken in order to visualize the micro-regions where the mapping was performed. As compared with the conventional SEM images presented in Figure 1a–f, these optical images with low depth of field, show regions that do not lie within the focal plane of the optics and are therefore not well resolved.

Selective Raman features such as the vibrational line of diamond at 1334 cm^−1^, the band at 1230 cm^−1^ attributed to boron incorporation into the diamond lattice [24], and the band around 1500 cm^−1^ corresponding to amorphous carbon impurities, were considered for performing the confocal Raman mapping images. For visualization, these three constituents were labeled using the following pseudo-colors: red for diamond, blue for BDD, and green for carbon impurities. An expected color trend from dominant red (Figure 2b), to blue (Figure 2f,h) is observed with increasing amounts of TMB rector feed gas. Besides a more rapid crystallization process induced by the changes in the growth factors such as substrate temperature and B-doping level, these Raman images make obvious that even at high doping levels there is a non-uniform, preferential incorporation of boron into the microstructure and grain-distribution characteristics of diamond; pure diamond crystallites (red color) can still be observed. The very sparse presence of C sp^2^ species increases with increasing B doping level and substrate temperature. This affirmation is supported by the Raman spectra of these samples that are presented in Figure 5 and further analyzed below; they include the integrated Raman spectra obtained for the entire 100 µm × 100 µm areas of these images.

**Figure 3:**
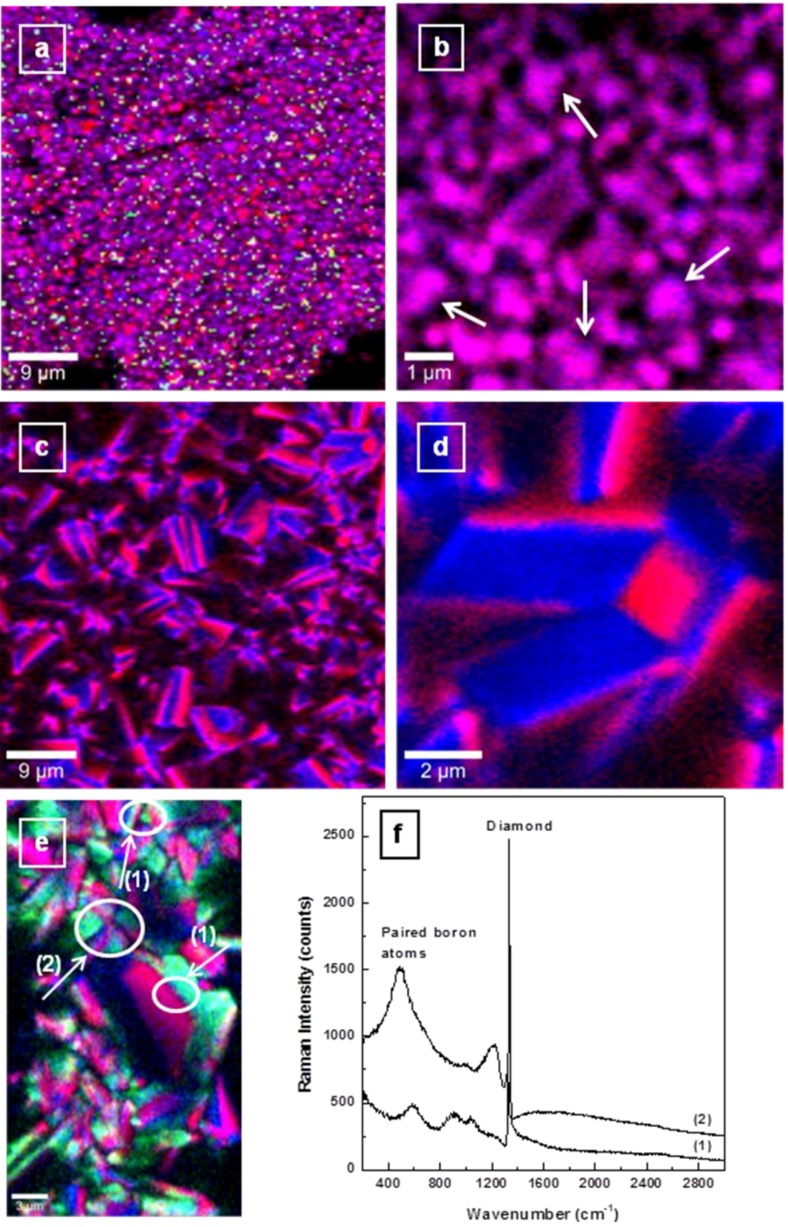
(**a**–**e**) Confocal Raman mapping images acquired at different resolutions for boron-doped diamond grown with 4.9, 10.8 and 12.5 ppm TMB feed gas; and (**f**) integrated Raman spectra in two different regions of Raman mapping image shown in (**e**) for a film grown with 12.5 ppm TMB addition. The spectra are vertically translated for clarity.

More evidence of the literature-predicted grain boundary accumulation of boron and its preferential incorporation [19,22,25] can be observed in Figure 3a–e, which includes the high-resolution Raman mapping images of BDD crystallites grown with the addition of 4.9, 10.8, and 12.5 ppm TMB. For consistency, the same pseudo-colors were used. A uniform purple color (combination of red and blue), which suggests a more even incorporation of boron into smaller crystallites, can be seen in Figure 3a,b for a boron doping level of 4.9 ppm TMB. However, an important aspect that should not be neglected is that well-defined growth directions are not yet completely developed for small crystallites, potentially leading to this visual local uniformity of boron incorporation.

While the presence of carbon impurities is considered in performing the Raman mapping at a larger scale (Figure 3a), for an easier visualization of the slight accumulation of boron at the grain boundaries, this option is eliminated for high-resolution Raman mapping (Figure 3b). Indeed, as marked by the solid arrows, boron atoms have the tendency of building up at crystallite boundaries, experimentally validating, for the first time, this theoretical prediction [19]. It has been suggested that this boron accumulation contributes to the overall decrease in the carrier mobility and material conductivity, as it might create alternative conductive pathways which compete with those of through-grain conduction [18,19,20].

An increase in substrate growth temperature, although it allows incorporation of a larger amount of boron without its visible accumulation at the grain boundaries, also induces preferential dopant insertion onto microcrystallite facets, as observed in the Raman mapping images presented in Figure 3c,d, which again were recorded without consideration of the presence of C sp^2^ species. The reason for this predicted effect is that during crystallization, different values of the surface specific energies are expected for different facets (e.g., 6.49 × 10^24^ J∙m^−2^∙mol^−1^ for {111}, 7.95 × 10^24^ J∙m^−2^∙mol^−1^ for {110}, and 1.124 × 10^25^ J∙m^−2^∙mol^−1^ for {100}) [12]. From theoretical calculations, a decrease in the surface specific energy of {111} facets is anticipated, favoring boron assimilation at these lattice points. In the cases of cubic {100}, dodecahedral {110}, and {113} surfaces, due to the existence of uncompensated bonds from boron atoms and to the differences in B–B and C–C bond lengths, this decrease in the specific energy is unfeasible [25]. Even if higher B-doping levels are considered and definitely achievable for microcrystalline structures, these Raman mapping images demonstrate that formation of a very thin and stable conductive boron layer from covalently bonded molecular boron networks is unlikely if a CVD growth process is employed. Instead, a random accumulation of paired boron atoms at the crystallite grain boundaries is still going to occur at excessive doping, limiting material conductivity. Increasing substrate growth temperature, while it augments crystallite sizes with potential formation of even a single crystal (*i.e.*, high-pressure high-temperature growth conditions), has the drawback of increasing the amount of unwanted carbon impurities (at high temperatures creation of a higher carbon flux from endothermic methane decomposition is expected). Direct proof of this statement can be seen in Figure 3e,f, where high-resolution Raman mapping of BDD films grown with 12.5 ppm TMB and the integrated Raman spectra in different regions of this image, respectively, are presented.

**Figure 4:**
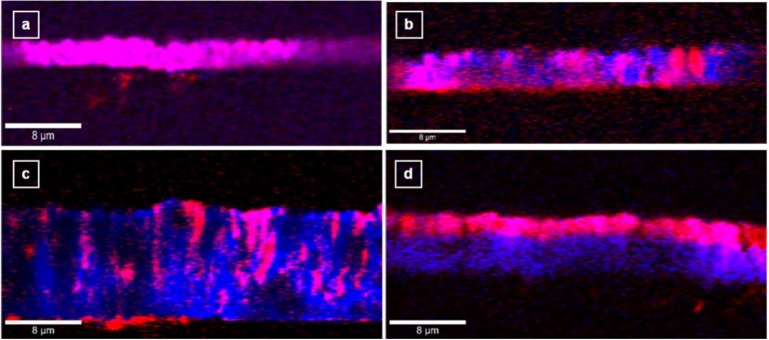
Side-wall Raman mapping images of boron-doped diamond thin films grown with different TMB feed gas amounts: (**a**) 4.9 ppm; (**b**) 7.4 ppm; (**c**) 10.8 ppm; and (**d**) sequentially doped with 2.5 ppm and 7.4 ppm TMB.

Besides characteristic columnar growth of polycrystalline microstructures (an observation consistent with the SEM images presented above), the side-wall Raman mapping images presented in Figure 4a–d show a non-uniform boron incorporation across the whole film thickness.

Again, if 4.9 ppm TMB feed gas is used for doping, a slightly improved homogeneity of boron distribution into the diamond lattice is observed (Figure 4a), while amounts of 7.4 and 10.8 ppm TMB (Figure 4b,c, respectively) induce preferential boron insertion and the existence of micro-regions of undoped diamond. The capability of confocal Raman mapping in detecting differences in doping levels in multilayer BDD thin films grown with different amounts of TMB is demonstrated in Figure 4d. It is obvious from this image that the top BDD layer has been grown with lower boron doping (2.5 ppm TMB) than the BDD layer underneath (7.4 ppm TMB).

**Figure 5:**
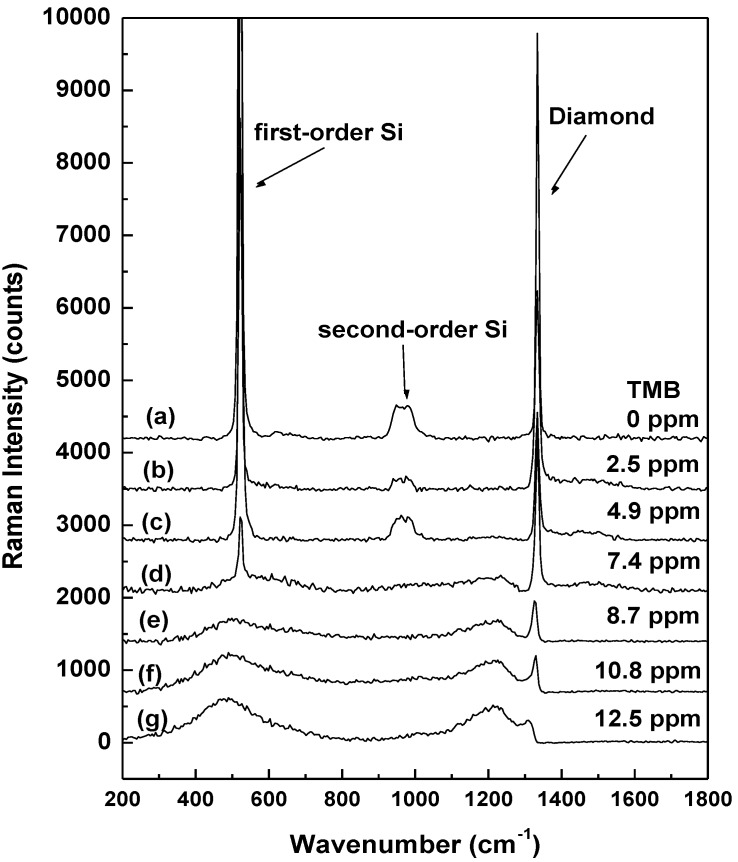
Raman spectra of undoped and boron-doped diamond samples grown with different TMB amounts, as labeled. The spectra are vertically translated for easier visualization.

The Raman spectra of undoped diamond (0 ppm TMB), and BDD thin films grown with increasing TMB concentration, are presented in Figure 5. To compensate for the observed inhomogeneity of each sample, besides the integrated Raman spectra of previously analyzed confocal Raman images, additional Raman measurements, each of an acquisition time of 5s per spectrum, were performed for each sample in different spots; the averages of all these Raman spectra are presented here. An appropriate background subtraction was performed.

A sharp Raman line at 1334 cm^−1^ attributed to diamond and two broad features centered around 500 and 1230 cm^−1^ are seen in the spectra labeled (d–g). Similar results have been reported in the literature [5,21,24,,25,27] and the origin of the last two bands was attributed, based on calculations of the phonon density of states, to the boron incorporation in the diamond lattice. Therefore, the observed increase in their intensities with the use of higher amounts of TMB feed gas during the growth process is anticipated. Since the 500 cm^−1^ band might be also associated with pairs of boron atoms [24,27], a stronger increase in the intensity of this band than in the intensity of the 1230 cm^−1^ feature also implies that only part of the total boron amount incorporated in the material contributes to the actual free carriers (hole) concentration, the rest aggregating at grain boundaries. The last Raman spectrum of this figure, spectrum (g), hints at this possibility. Doping levels ranging between 10^20^ and 10^21^ B/cm^3^ are obtained by fitting the Raman peak at ~500 cm^−1^ with a combination of Gaussian and Lorentzian line shapes in the spectra (d–g) and by using the relationship [27]:

[B] (in cm^−3^) = 8.44 × 10^30^ exp (−0.048 ω) (1)

where ω is the position of the peak in cm^−1^.

The position and sharpness of the 1334 cm^−1^ peak near the standard signature of diamond at 1332 cm^−1^, with FWHM ranging between 3.5 and 5.8 cm^−1^ for TMB ranging from 0 to 7.4 ppm, implies that no significant compressive stress has been induced in the material during the growth process. An increasing asymmetry of this zero phonon line and a decrease in its intensity is observed with increasing doping level. This asymmetry is known to be due to Fano-type interference between the discrete zone-center phonon and the continuum electronic states and marks the onset of metallic-like conductivity [21,28]. Heavily doped samples are illustrated by the spectra (e–g). The peak at 521 cm^−1^ and the band centered at 970 cm^−1^ correspond to the first and second-order scattering from the Si substrate, respectively. The visible decrease in the intensities of these vibrational lines of Si to their complete disappearance at higher boron doping level is consistent with a likely increase in the optical opacity of the BDD material.

**Figure 6:**
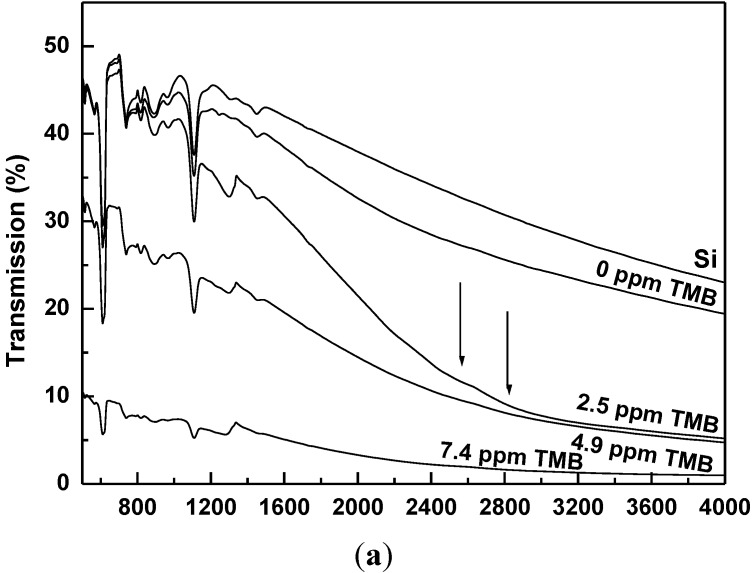

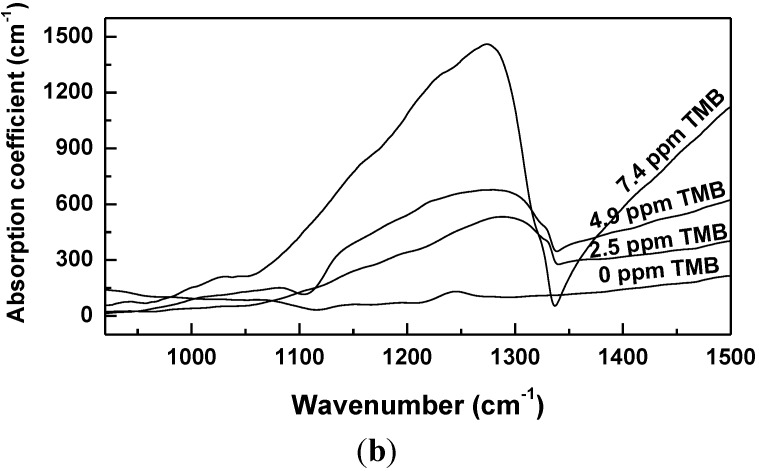
(**a**) Transmission spectra in the mid-infrared region; and (**b**) absorption coefficient spectra around and including 160 meV (1290 cm^−1^) of boron-doped diamond samples grown with different TMB feed gas amounts, as labeled.

The absorption properties of the samples were investigated by mid-infrared transmission measurements and the results are presented in Figure 6a. Saturation effects in absorption prohibited spectral measurements of samples grown with more than 7.4 ppm TMB.

While the spectrum of the undoped sample (0 TMB) has a trend very similar to that of the Si substrate spectrum, simply demonstrating diamond transparency in this spectral region without visible evidence of boron incorporation, there is a very abrupt decrease in the transmission spectrum of the sample doped with 2.5 ppm TMB. This relatively strong absorption is caused by two very inconspicuous and broad features around 2500 and 2800 cm^−1^ corresponding to excited states of bounded holes and marked with solid arrows. In addition to the characteristic Si vibrational lines, all the spectra show the presence of the boron-induced one phonon absorption band around 1290 cm^−1^ (160 meV). An estimation of the boron content can be achieved from the coefficient of absorption of this band by using the relationship [13]:

*N*_A_ − *N*_D_ = (1.6 × 10^17^) ∆α_1290_ ± 20% (2)

where *N*_A_ and *N*_D_ are the numbers of substitutional acceptors and donors, respectively. Values of uncompensated boron acceptors ranging from 8 × 10^18^ to 2 × 10^2^^0^ atoms/cm^3^ are obtained from the corresponding absorption coefficients at 1290 cm^−1^, spectra for which are presented in Figure 6b. The intrinsic Si substrate absorption was considered and appropriately subtracted from these spectra. Since the critical percolation threshold of metallic-like conductivity starts at approximately 10^2^^0^ atoms/cm^3^, these values corroborate with the outcomes of the Raman measurements, where Fano-type interference is observed for these samples.

### 3.3. Dopamine Sensing with FSCV

**Figure 7:**
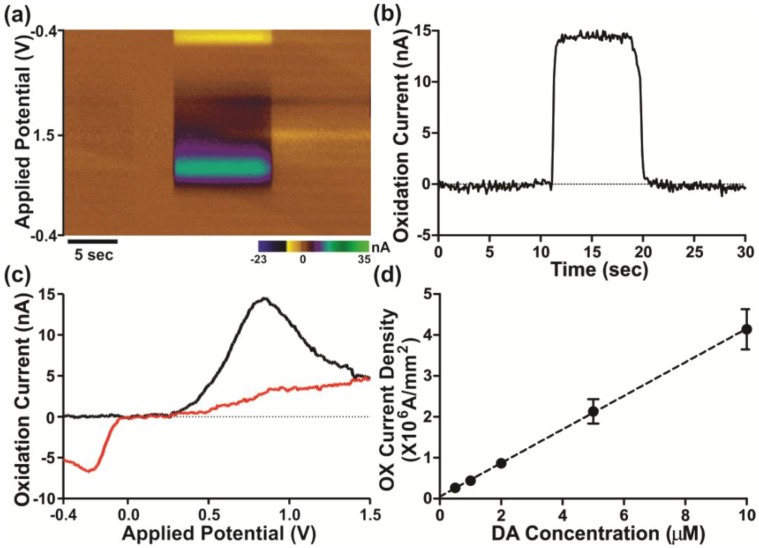
Dopamine (DA) detection with fast-scan cyclic voltammetry (FSCV). (**a**) Representative FSCV color plot showing DA addition (5 μM) to an *in vitro* flow cell analytical system. The *x*-, *y*-, and color gradient axes represent time, the applied voltage, and the resulting current changes, respectively; (**b**) current *versus* time plot of the DA oxidation peak potential at +0.8 V; (**c**) cyclic voltammogram obtained following injection of 5 µM DA in (**a**). Black and red lines indicate forward (−0.4V → +1.5V) and reverse-going (+1.5V → −0.4V) current responses to the triangular potential protocol, respectively; and (**d**) oxidation (OX) current density *versus* DA concentrations showing linear response of the diamond electrode.

To confirm the functionality of a BDD electrode that has been grown using about 5 ppm TMB feed gas, we performed FSCV of dopamine. A triangular waveform of potential between −0.4 V and +1.5 V was applied to the BDD electrode. Plots of the signal current responses to the applied potentials are shown in Figure 7a–c.

As observed in Figure 7a,c, dopamine (DA) produces current *vs.* voltage curves (voltammograms) that are distinguishable based on its oxidation and reduction current peaks. Since the fast scan rate generates a large background current, which can obscure the Faradaic current of the analyte of interest, a background-subtraction technique has been applied to the voltammogram presented in Figure 7c. The peaks of DA oxidation and reduction currents (pseudo-colored in Figure 7a by green and yellow, respectively) were each recorded with reference to an Ag/AgCl electrode at +0.8 V and −0.2 V, respectively. While a typical DA oxidation peak appears at +0.6 V with a carbon fiber electrode [29,30], a consistent positive shift of 0.2 V was observed for the oxidation peak potential of a diamond electrode. Further monitoring of the performance of the BDD electrode for analyte detection is presented in Figure 7b, where the peak oxidation current of DA (5 μM) is plotted *vs.* time. Also, as seen in Figure 7d, the detected current density of the oxidation peak current increases linearly with the increment of DA concentrations in the range of 0.5 to 10 μM, with a correlation coefficient of 0.99 based upon four trials, demonstrating the reproducibility of the BDD electrode performance in DA detection.

## 4. Conclusions

With the research goal of discovering ways to improve the quality of BDD thin films grown by CVD for further use in FSCV electrode coating, we note that, according to our spectroscopic results, improvement is achievable if a moderate doping that avoids grain boundary aggregation is employed. Although this material has been amply studied, the proper boron doping amount that will increase its conductive properties is still not completely evident. Notably, the multifaceted aspects that should be considered in its qualitative evaluation such as the growth conditions (which include substrate temperature and type as well as precursor gases and amounts) and the chemical reaction mechanisms (which vary from case to case depending on the particular application) lead to some apparent inconsistency in the literature.

The experimental investigations reported here prove the effectiveness of confocal Raman mapping in visually depicting small structural modifications in the morphology of boron-doped diamond thin films and in providing valuable information that is complementary to that obtained by IR absorption spectroscopy and by other means of electron microscopy. It also validates theoretical predictions such as dopant accumulation at the grain boundaries and its preferential incorporation into the diamond lattice. Since these effects negatively contribute to material conductive properties, for applications such as FSCV, where uniform doping of BDD thin films is required only over micrometer domains (only at the tip of the electrode), this latter characteristic could be realized in the current research by using ~5 ppm TMB feed gas during the growth process. For this amount of boron precursor, a dopant content of about 10^20^ B/cm^3^, which is a borderline value for metallic-like conductivity, is derived from both our Raman and infrared absorption spectral analyses. However, since not all B atoms are going to contribute to material conductivity, the number of free carriers is less than this value. The alternative solution of increasing the doping level, although achievable as demonstrated in this work, is not going to enhance desired material characteristics, largely because of accumulation of dopant and C sp^2^ impurities at crystallite boundaries.

The FSCV measurements presented in this study corroborate with the microscopic and spectroscopic analysis, demonstrating that under appropriate growth conditions, BDD electrodes can detect DA in the range of 0.5–10 μM with high reproducibility. Even though more work needs to be done for developing high quality diamond-based materials for use in bio-medical applications, the results of this research show the usefulness of a multi-technique analysis approach (*i.e*., confocal Raman mapping and voltammetry) in supplying comprehensive information valuable to accomplishing this purpose.
